# Supplementary material for: Impact of Sea Warming and 17-α-Ethinylestradiol Exposure on the Lipid Metabolism of Ruditapes philippinarum Clams
Source: Int J Mol Sci. 2023 May 30;24(11):9485. doi: 10.3390/ijms24119485 (PMC10253961; doi:10.3390/ijms24119485)
Supplement: Supplementary file 1 [file ijms-24-09485-s001.zip › ijms-2426259-supplementary.pdf]

Supplementary Information

Impact of Sea Warming and 17-α-Ethinylestradiol Exposure on the Lipid Metabolism of *Ruditapes philippinarum* Clams

João A. Rodrigues [1,†], Daniela S. C. Bispo [1,†], Mónica G. Silva [2], Rita Araújo [1], Amadeu M. V. M. Soares [2], Rosa Freitas [2] and Ana M. Gil [1,*]

[1] CICECO–Aveiro Institute of Materials, Department of Chemistry, University of Aveiro, Campus Universitário de Santiago, 3810-193 Aveiro, Portugal

[2] Department of Biology & Centre for Environmental and Marine Studies (CESAM), University of Aveiro, Campus Universitário de Santiago, 3810-193 Aveiro, Portugal

* Correspondence: agil@ua.pt; Tel.: +351-234370707

† These authors contributed equally to this work.

Figure S1. Schematic representation of the experiment of bivalves' exposure to EE2, at 17 °C and 21 °C.

Figure S2. Sequential pairwise PLS-DA score plots obtained for the ^{1}H NMR spectra of bivalves' lipidic extracts upon EE2 exposure at 17 ºC (left, circles) and 21 ºC (right, diamonds).

Table S1. Features of pairwise PLS-DA models either relative to sequential comparisons of groups subjected to gradually increasing EE2 concentrations (top section) or compared to controls (bottom section).

Table S2. Correlation values ($|r| > 0.50$) in bivalves exposed to different EE2 concentrations, at 21 °C.

Figure S1. Schematic representation of the experiment of bivalves' exposure to EE2, at 17 °C and 21 °C. After acclimation, samples were exposed to different concentration levels of EE2: 0 ng/L (control), 5, 25, 125 and 625 ng/L. For each concentration level and temperature, 12 samples were considered: 4 individuals per aquarium and 3 aquaria at each temperature (total of 60 samples/temperature). After 28 days of exposure, an extraction procedure was followed, using a methanol (MeOH): chloroform ($CHCl_3$): water (H_2O) method [47], obtaining a polar and a lipidic extract, the latter following for NMR analysis. [a] polar extract analysis reported in [14].

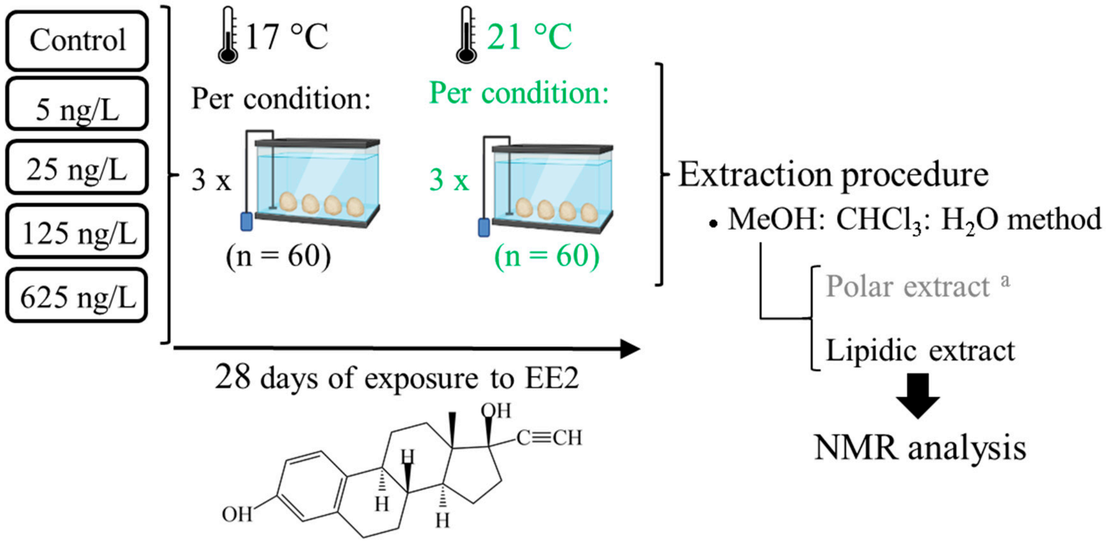

Figure S2. Sequential pairwise PLS-DA score plots obtained for the ^{1}H NMR spectra of bivalves' lipidic extracts upon EE2 exposure at 17 ºC (left, circles) and 21 ºC (right, diamonds): a) 5 ng/L (orange) vs. 0 ng/L (red), b) 25 ng/L (lighter green) vs. 5 ng/L (orange), c) 125 ng/L (darker green) vs. 25 ng/L (lighter green) and d) 625 ng/L (blue) vs. 125 ng/L (darker green). Abbreviations: R^2X: fraction of the variation of X-variables explained by the model; R^2Y: fraction of the variation of Y-variables explained by the model; Q^2, predictive power (≥ 0.5 for robust models).

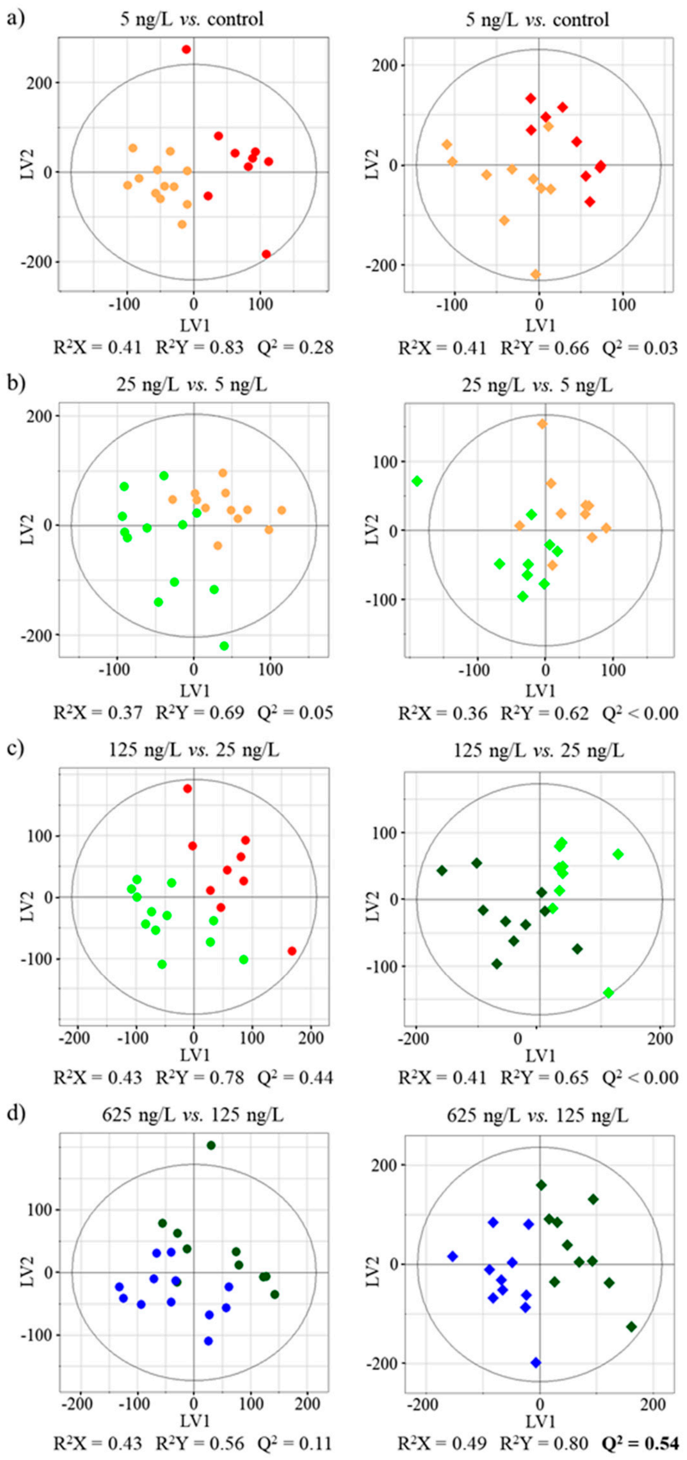

Table S1. Features of pairwise PLS-DA models either relative to sequential comparisons of groups subjected to gradually increasing EE2 concentrations (top section) or compared to controls (bottom section). Abbreviations: R^2X: fraction of the variation of X-variables explained by the model (high for robust models); Q^2, predictive power (values in bold and underlined are ≥ 0.5).

**Sequential PLS-DA models for increasing EE2 concentrations**								
	5 ng/L vs. 0 ng/L		25 ng/L vs. 5 ng/L		125 ng/L vs. 25 ng/L		625 ng/L vs. 125 ng/L	
Temperature	R^2X	Q^2	R^2X	Q^2	R^2X	Q^2	R^2X	Q^2
17 °C	0.41	0.28	0.37	0.05	0.47	0.03	0.43	0.11
21 °C	0.41	0.03	0.36	< 0.00	0.41	< 0.00	0.49	**<u>0.54</u>**
**PLS-DA models for each exposure EE2 concentration vs. controls**								
	5 ng/L vs. 0 ng/L		25 ng/L vs. 0 ng/L		125 ng/L vs. 0 ng/L		625 ng/L vs. 0 ng/L	
Temperature	R^2X	Q^2	R^2X	Q^2	R^2X	Q^2	R^2X	Q^2
17 °C	0.41	0.28	0.43	0.44	0.41	0.05	0.39	**<u>0.52</u>**
21 °C	0.41	0.03	0.28	< 0.00	0.33	0.10	0.46	**<u>0.51</u>**

Table S2. Correlation values (|*r*| > *0.50*) in bivalves exposed to different EE2 concentrations, at 21 °C obtained for triglycerides (TGs) vs. saturated FAs $(CH_2)_n$; phosphatidylcholine (PtdCho) vs. phosphatidylethanolamine (PtdEtn); PtdCho vs. polyunsaturated fatty acids (PUFAs); PUFAs vs. glycine; PUFAs vs. taurine.

**Temperature**	**5 ng/L vs. 0 ng/L** ***r***	**25 ng/L vs. 0 ng/L** ***r***	**125 ng/L vs. 0 ng/L** ***r***	**625 ng/L vs. 0 ng/L** ***r***
**TGs vs. saturated FAs**				
17 °C	---	---	---	---
21 °C	0.70	---	0.70	0.67
**PtdCho vs. PtdEtn**				
17 °C	0.69	0.62	0.72	0.73
21 °C	0.58	0.72	0.59	0.66
**PtdCho vs. PUFAs**				
17 °C	0.89	0.91	0.88	0.93
21 °C	0.86	0.81	0.86	0.85
**PUFAs vs. glycine**				
17 °C	---	---	---	---
21 °C	0.73	0.74	0.63	0.76
**PUFAs vs. taurine**				
17 °C	---	---	---	---
21 °C	---	---	---	0.52
